# Miniaturized Electrochemical Sensors to Monitor Fetal Hypoxia and Acidosis in a Pregnant Sheep Model

**DOI:** 10.3390/biomedicines9101344

**Published:** 2021-09-28

**Authors:** Míriam Illa, Laura Pla, Sergio Berdún, Mònica Mir, Lourdes Rivas, Samuel Dulay, Nicole Picard-Hagen, Josep Samitier, Eduard Gratacós, Elisenda Eixarch

**Affiliations:** 1 BCNatal | Fetal Medicine Research Center (Hospital Clínic and Hospital Sant Joan de Déu), Universitat de Barcelona, 08028 Barcelona, Spain; laura.pla.codina@gmail.com (L.P.); sberdun@pcb.ub.es (S.B.); egratacos@sjdhospitalbarcelona.org (E.G.); EIXARCH@clinic.cat (E.E.); 2 Institut de Recerca Sant Joan de Déu, 08950 Esplugues de Llobregat, Spain; 3 Centro de Investigación Biomédica en Red en Bioingeniería, Biomateriales y Nanomedicina (CIBER-BBN), Monforte de Lemos 3–5, Pabellón 11, 28029 Madrid, Spain; mmir@ibecbarcelona.eu (M.M.); jsamitier@ibecbarcelona.eu (J.S.); 4 Nanobioengineering Group, Institute for Bioengineering of Catalonia (IBEC), Baldiri Reixac 15-21, 08028 Barcelona, Spain; lrivas@ibecbarcelona.eu (L.R.); dulaysamuel@yahoo.com (S.D.); 5 Department of Electronics and Biomedical Engineering, University of Barcelona, Martí i Franquès 1, 08028 Barcelona, Spain; 6 ToxAlim (Research Centre in Food Toxicology), Université de Toulouse, INRAE, ENVT, INP-Purpan, UPS, 31027 Toulouse, France; nicole.hagen@envt.fr; 7 Institut d’Investigacions Biomèdiques August Pi i Sunyer (IDIBAPS), 08028 Barcelona, Spain; 8 Centre for Biomedical Research on Rare Diseases (CIBER-ER), 08028 Barcelona, Spain

**Keywords:** umbilical cord occlusion, continuous monitoring of acid-base status, high-risk pregnancies, electrochemical sensors

## Abstract

Perinatal asphyxia is a major cause of severe brain damage and death. For its prenatal identification, Doppler ultrasound has been used as a surrogate marker of fetal hypoxia. However, Doppler evaluation cannot be performed continuously. We have evaluated the performance of a miniaturized multiparametric sensor aiming to evaluate tissular oxygen and pH changes continuously in an umbilical cord occlusion (UCO) sheep model. The electrochemical sensors were inserted in fetal hindlimb skeletal muscle and electrochemical signals were recorded. Fetal hemodynamic changes and metabolic status were also monitored during the experiment. Additionally, histological assessment of the tissue surrounding the sensors was performed. Both electrochemical sensors detected the pO_2_ and pH changes induced by the UCO and these changes were correlated with hemodynamic parameters as well as with pH and oxygen content in the blood. Finally, histological assessment revealed no signs of alteration on the same day of insertion. This study provides the first evidence showing the application of miniaturized multiparametric electrochemical sensors detecting changes in oxygen and pH in skeletal muscular tissue in a fetal sheep model.

## 1. Introduction

Perinatal asphyxia is a major cause of severe brain damage and death during the perinatal period. While acute asphyxia affects 1 in every 1000 live births in developed countries [1], chronic forms of fetal hypoxia may occur in up to 1 to 3% of pregnancies. The main causes of chronic fetal hypoxia are complications related to placental insufficiency, namely preeclampsia and fetal growth restriction. Irrespective of the cause, chronic hypoxia generally results from insufficient oxygen supply to the fetus, eventually leading to hypoxia, hypercapnia, and acidosis. In a primary phase, fetal circulatory and non-circulatory adaptive mechanisms try to cope with asphyxia and preserve vital organ function. However, in severe and/or prolonged insults, compensatory mechanisms fail, leading to cell death via necrosis and apoptosis [2].

Close monitoring of pregnancies with placental insufficiency is based on Doppler evaluation of fetal hemodynamics [3]. Changes in the hemodynamics of fetal blood flow in the ductus venosus or in the umbilical artery are highly suggestive of progressive hypoxia and eventually of fetal acidemia [4], and Doppler is used to decide elective delivery when the risk of fetal death or brain damage is deemed too high [5]. Since fetuses with severe forms of placental insufficiency are normally extremely premature, timing of delivery is critical to avoid fetal death but prolong pregnancy as much as possible to minimize the neonatal complications of prematurity.

While Doppler ultrasonography has been an invaluable tool to improve the timing of delivery in critically ill, very preterm fetuses, it is still limited by the impossibility of performing continuous monitoring. The development of a system to monitor acid-base status continuously would be a great step forward in the clinical management of very high-risk pregnancies. Previous research has demonstrated the usefulness of oxygen and pH sensors to monitor acid-base status continuously in different tissues. Oxygen sensors based on either pulse-oximetry or fiber-optic sensing inserted in fetal tissue during labor in humans or placed endovascularly during endoscopic fetal surgery in sheep have shown promising results in detecting oxygen variations [5,6,7,8]. Similarly, sensors able to monitor the levels of fetal pH have also been described [8,9,10,11]. Despite all these advances, these sensors are not suitable for insertion in early pregnancy, due to the need to protect the uterus and the very small size of fetal tissues. In this regard, miniaturized electrochemical sensors insertable through a needle would be required in order to monitor fetal acid-base status. Previous studies from our group have demonstrated that miniaturized electrochemical sensors could monitor oxygen or pH changes continuously in a model of ventilatory hypoxia in adult rabbits [12,13,14]. The development of a multiparametric electrochemical sensor aiming to monitor oxygen and pH at the same time would offer a great advantage in the monitoring of these pregnancies.

In this study, we aimed to test the performance of integrated and miniaturized pH and oxygen sensors to enable implantation for fetal monitoring and to detect changes in these parameters simultaneously in a lamb model of acute hypoxia [15,16]. We have used a sequential UCO to induce fetal acid-based metabolite alterations in a gradual and controlled manner and the electrochemical sensor signals, blood test analyses, and Doppler hemodynamics were analyzed. 

## 2. Materials and Methods

### 2.1. Animals: Ethics, Housing, and Preparation

A total of eight Ripollesa pregnant ewes with gestational ages between 115–125 days (term = 147–150 days) and weight ranging from 60 to 80 kg were included in this study. Five of the eight ewes carried singletons and three of the eight carried twins. The animals were provided by a certified commercial farm (Animal Bianya, Catalonia, Spain) and were acclimated for two weeks before surgery. Their clinical condition was assessed by veterinary staff before starting the study. The animals were housed following local standards. Ewes were food fasted 24 h before the surgery and water fasted 12 h before the surgery. Animal handling and all experimental procedures were performed in accordance with applicable regulations and guidelines and with the approval of the Animal Experimental Ethics Committee of the Universitat de Barcelona (Ref. 214.17) and the competent authority Generalitat de Catalunya (Ref. 9645).

### 2.2. Miniaturized Electrochemical Sensors: pH and pO_2_

The sensors used in this article were developed and optimized in previous work [13,14]. In previously reported articles, the sensitivity, specificity, reproducibility, and response time of these sensors are described, which were validated in adult rabbits. In the present work, difficulties in fetal monitoring are faced, such as the smaller size for the insertion of sensors, more instability in vivo, lower oxygen content in fetal blood, and lower changes of analytes in tissue after ischemia. For fetal application, the sensors were fabricated to be longer (1 m). For that reason, the insulating layer of one end of the metallic wires (Pt or Ag, 15 cm in length and 125 µm in diameter) was carefully removed to solder it with a copper wire (1 m in length). Once the microsoldered section had been isolated, the modifications for preparing the working and the reference electrodes for both types of electrochemical sensor were performed as previously described [13,14]. After the sensors were individually functionalized, they were integrated into a biocompatible tube with a final diameter of 500 µm (See supporting information Figure S1).

### 2.3. Animal Instrumentation

#### 2.3.1. Ewe Surgery 

The animals were premedicated with ketamine, xylazine, and midazolam (4 mg/kg, 0.2 mg/kg, and 0.2 mg/kg respectively, IM). For induction anesthesia, 2–4 mg/kg (IV) of propofol was administered. An endotracheal tube was then inserted, and mechanical pressure-controlled ventilation was started. Anesthesia was maintained with isoflurane (1.5–3%) using a semi-closed system. Heart rate, respiratory rate, oxygen saturation, end-tidal carbon dioxide, and reflexes were monitored every 15–30 min. Body core temperature was maintained using an electric pad. Before the start of surgery, 1 L of saline 0.9% NaCl was infused using a jugular catheter, and buprenorphine (0.02 mg/kg, IV), and progesterone (150 mg, SC) were also administered. Urinary tract cannulation and orogastric intubation were performed. 

Before the start of the surgery, the animals were prepared for aseptic surgery and 10 mL of lidocaine (2%) were injected intradermally on the site of the abdominal incision. A 15-cm infraumbilical midline laparotomy was performed for uterus exteriorization. The number of fetuses, their position, the fetal heart rate (FHR), and deepest vertical pocket (DVP) of amniotic fluid were recorded using ultrasonography (VIVIQ Q, lineal probe, 7.5 MHz) before hysterotomy. In twin gestations, only one uterine horn was exposed and used for the experiment. The fetal position was identified and a 6–8 cm hysterotomy was performed using a running suture on both incisional sides for fetal membrane fixation (poliglecaprone, 2/0). During the surgery, fluidotherapy with saline 0.9% NaCl solution was administered (500 mL/h) and serial maternal gasometry from the jugular vein (between 2 and 4 samples per experiment) was performed throughout the experiment in order to evaluate maternal acid-base status (EPOC reader and EPOC BEGM test card, Alere/Siemens healthcare, Barcelona, Spain).

#### 2.3.2. Fetal Instrumentation 

Fetal hindlimbs were exteriorized through hysterotomy, and fentanyl (0.2 mL, IM) was administered for fetal anesthesia. An incision of 3–4 cm in the right inguinal zone was performed for the fetal iliac artery dissection. Catheterization of the iliac artery was performed for all the included fetuses (*n* = 8) with a thin catheter (0.75 mm of internal diameter and 1.45 mm of external diameter) and was used for serial fetal blood sampling in order to evaluate acid-based metabolites (using the EPOC reader and EPOC BEGM test card, Alere/Siemens Healthcare, Barcelona, Spain). The catheter was fixed with simple knots (silk, 3/0) and fetal skin was closed with a running suture (polyglycolic acid, 3/0). Thereafter, 1–2 oxygen and 1–2 pH electrochemical sensors were inserted in the right and left femoral fetal quadriceps, respectively. We did a 1-cm skin incision and the femoral quadriceps was exposed. Electrochemical sensors were implanted through a 2–3 mm muscle incision and secured to the parenchyma using simple knots (silk, 3/0). One to two oxygen or pH electrochemical sensors were inserted in each animal. The skin was closed using a running suture (polyglycolic acid, 3/0). Afterwards, the umbilical cord was also exteriorized through hysterotomy and a vascular occluder (OC20HD, UNO Roestvaststaal BV, Zevenaar, Netherlands) connected to a 10 mL syringe loaded with saline was placed around the umbilical cord. Finally, fetal hind limbs and umbilical cord were interiorized inside the uterus. An amniotic catheter was also inserted through the hysterotomy in order to infuse warm NaCl 0.9% to maintain the temperature and amniotic volume throughout the experiment. The amniotic cavity insufflation was performed until the initial DVP was reached (approximately 150 mL). Sensor wires, iliac vascular catheter, amniotic catheter, and the occluder tubing were exteriorized through the uterine incision. Hysterorraphy was then performed using a running suture (polidioxanone, 3/0). The electrodes of the electrochemical sensors were connected to a portable electrochemical device for the continuous monitoring of the signals of the oxygen and pH electrochemical sensors. During the experiment, the uterus was kept outside the abdominal cavity and was continuously rinsed with warm NaCl 0.9% and temperature controlled with an external thermal lamp.

### 2.4. Occlusion Protocol and Monitoring 

The occlusion protocol was divided into four different stages: (1) basal; (2) 50% occlusion; (3) 100% occlusion; and (4) recovery. For the basal and recovery stages, the occluder was deflated and for the occlusion periods, the occluder was inflated with 1.5 mL or 3 mL of saline in order to obliterate 50% or 100% of the umbilical cord, respectively, and reduce the blood flow. In each stage, fetal blood sampling and electrochemical sensor signals (oxygen and pH) were recorded at the same moment in 5-to-10-min intervals. For blood sampling analyses, 0.2 mL of blood from the fetal iliac artery catheter was obtained for fetal gasometry with EPOC system including pO_2_, pH, bicarbonate, potassium, and lactate. Additionally, fetal Doppler evaluation was also recorded in each stage using VIVID Q ultrasound equipment (GE Healthcare, Chalfont Saint Giles, UK) including (i) FHR; (ii) pulsatility index (IP) of the umbilical artery (UAPI) in the perivesical portion; (iii) IP of the ductus venosus (DVPI); (iv) maximum blood flow velocity (cm/s) in the umbilical vein (UV) and diameter (mm) of the UV in the intrabdominal portion of the vessel. For each stage, at least one measurement was obtained in each period. 

#### 2.4.1. Basal Stage

After finalizing fetal instrumentation and hysterorraphy, basal registration was then started for 10 min. During that time, two measurements of fetal and maternal gasometry and fetal sensor signaling (in nA from the oxygen sensor and mV from the pH sensor) were recorded. 

#### 2.4.2. Occlusion Stage

The 50% occlusion stage lasted 20 min, while the 100% occlusion stage lasted 10 min. During these periods, regular fetal gasometry was performed every 10 min in the 50% occlusion stage and every 5 min in the 100% occlusion stage. At the same time, sensor signaling (in nA from the oxygen sensor and mV from the pH sensor) was also recorded. 

#### 2.4.3. Recovery Stage 

After 10 min of 100% occlusion, the occluder was deflated and the recovery stage was started, lasting 20 min. Fetal gasometry and electrochemical sensor recording was obtained every 10 min during this stage.

### 2.5. Sampling and Histological Analyses

**Figure 1:**
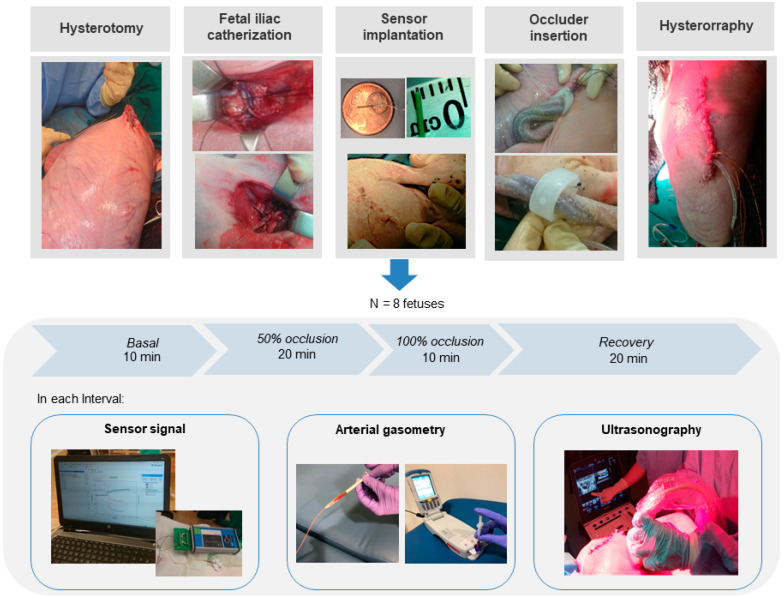
Illustrative images of the study design.

After the occlusion protocol, the animals were sacrificed with pentobarbital (200 mg/kg, IV) administrated to the ewe through the jugular vein. Death was confirmed by the cessation of circulation and breathing in both ewes and fetuses. Muscular tissue surrounding the insertion area of the electrochemical sensors inserted was carefully excised; fixed for one week by immersion in 10% buffered formalin and embedded in paraffin for further histological analyses. Paraffin blocks were serially cut in 5 μm thick transverse sections with a microtome and standard hematoxylin/eosin (Mayer’s Hematoyxlin, Ref. 51275 Sigma-Aldrich, Saint Louis, MO, USA; Eosin, Ref. 1.15935.0100 Merk, Darmstadt, Germany) and masson trichrome staining (Ref. HT15-1KT, Sigma-Aldrich, Saint Louis, MO, USA) were performed. One representative section from each sample was examined under an optic microscope (CH-9435, type DFC425C, Leica Microsystems, Hospitalet de Llobregat, Spain). Tissue integrity and cell infiltration were analyzed with hematoxylin eosin staining, whereas masson trichome staining was used for the evaluation of fibrotic reaction. The design of this study is summarized in Figure 1.

### 2.6. Statistical Analyses

The data were expressed as mean and standard error of the mean (SEM). A one-way ANOVA analysis with a Dunnett’s post-test was used for the analysis of the evolution of acid-based metabolite results, sensor parameters, and Doppler results during the UCO. Additionally, the association between electrochemical sensing results with the pO_2_ and pH values and Doppler results was analyzed by means of Pearson’s correlation analysis. Statistical significance was declared at *p* < 0.05 in all variables evaluated. All statistical analysis was performed using GraphPad Prism 6.0 software (San Diego, CA, USA).

## 3. Results

### 3.1. Acid-Based Metabolite Results 

Of the eight fetuses included in the study, one was excluded due to anesthesia problems that led to maternal acidosis during the basal period (maternal pH of 7.16). No other surgical or anesthesia complications were observed, leaving a final sample of seven fetuses included in the study. Maternal gasometry of the included animals was within normal ranges during all the different stages of the experiment. 

**Figure 2:**
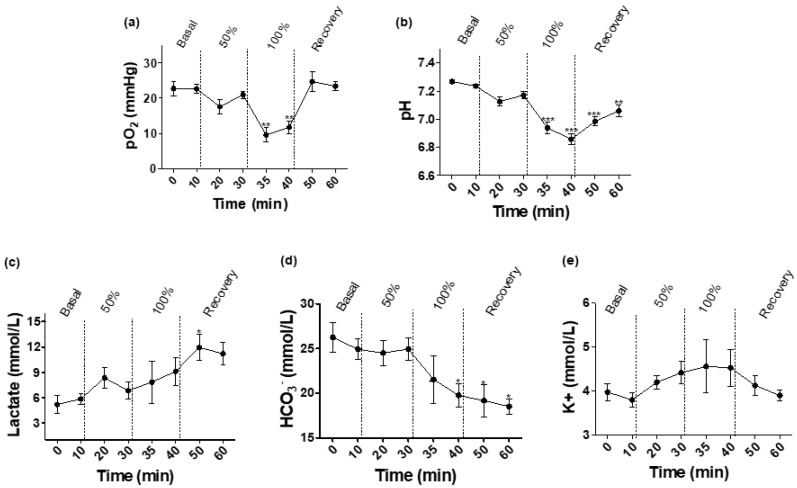
Evolution of arterial acid-based metabolites during the occlusion protocol (basal, 50% occlusion, 100% occlusion and recovery periods): (**a**) Partial pressure of oxygen (pO_2_); (**b**) pH; (**c**) lactate; (**d**) bicarbonate (HCO_3_^−^); and (**e**) potassium (K+) concentration. Data are expressed as mean ± SEM. Statistical significance was declared when * *p* < 0.05, ** *p* < 0.01, *** *p* < 0.001 between basal and each time point; *n* = seven animals, except for recovery period, *n* = four animals.

A description of the metabolite results obtained by fetal blood sampling during the different phases of the study is detailed in Figure 2. During the hypoxia-acidosis periods, a significant decrease in pO_2_, pH, and bicarbonate and a significant increase in lactate and potassium concentration in comparison with the basal period were observed. The pO_2_ decrease was the quickest and presented a more pronounced change in comparison with the rest of the metabolites, which presented more progressive and less marked changes. The changes were more marked and quicker in the 100% occlusion period. Finally, in the recovery stage, three of the seven fetuses did not overcome the fetal hypoxia-acidosis changes induced by the UCO and died during the recovery period. In these fetuses, the pH ranged between 6.69 and 6.77. The rest of the fetuses presented trends to an increase in pH levels without reaching the basal values (7.07). In the surviving animals, pO_2_ and K+ were the only parameters in the recovery phase that reached similar levels to those reported in the basal period.

### 3.2. Electrochemical Sensors

#### 3.2.1. pO_2_ Electrochemical Sensors

**Figure 3:**
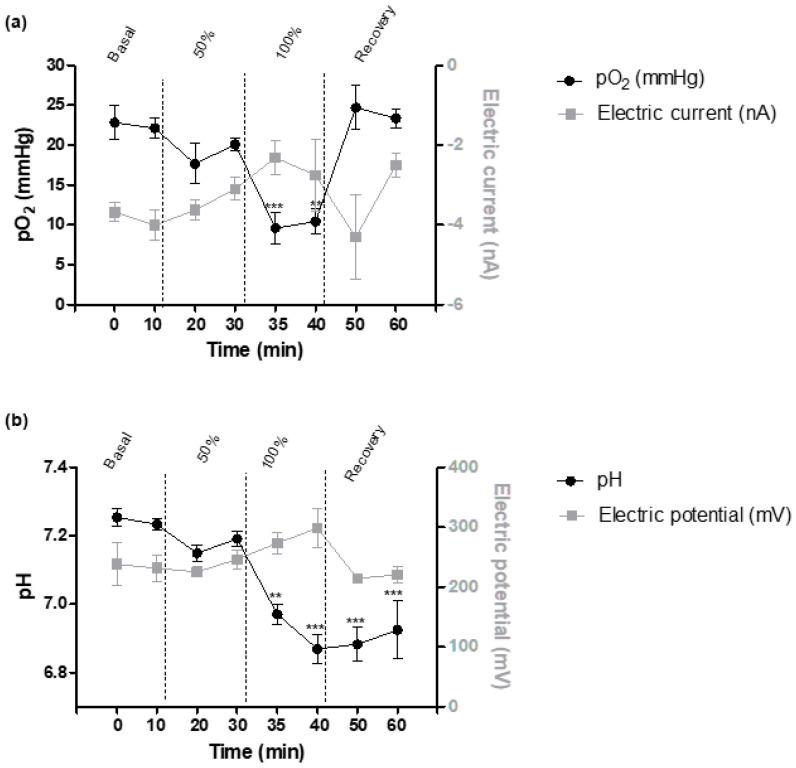
(**a**) Evolution of partial pressure of oxygen (pO_2_) (measured by arterial gasometry) and electric current (nA) (measured by oxygen electrochemical sensors); (**b**) evolution of pH (measured by arterial gasometry) and electric potential (mV) (measured by pH electrochemical sensors) during the occlusion protocol. Data are expressed as mean ± SEM. For pO_2_ sensing, nine electrochemical sensors; for pH sensing, 10 electrochemical sensors. Statistical significance was declared when ** *p* < 0.01 and *** *p* <0.001 between basal and each time point.

A total of nine pO_2_ electrochemical sensors were included for the final analysis. A description of the signal detected by the pO_2_ electrochemical sensor during the different stages of the study is detailed in Figure 3a. In the 50% occlusion stage, the electric signal presented an increase that was more marked in the 100% occlusion stage with an overall signal increase at the end of the 100% occlusion phase of 42.31% that was similar to the pO_2_ decrease observed with standard gasometry (56.57% decrease). In the recovery stage, during the first 10 min, the sensor signal decreased following the pO_2_ change observed by the standard EPOC equipment in this stage, reaching similar values of the basal period. 

#### 3.2.2. pH Electrochemical Sensors

A total of 10 pH electrochemical sensors were included for pH recordings in tissue. A description of the signal detected by the pH electrochemical sensor during the different stages of the study is detailed in Figure 3b. In the 50% occlusion stage, the electric signal presented an increase that was more marked in the 100% occlusion stage with an overall signal increase of 28.67% that was higher than pH change shown by EPOC (5.03% of decrease). In the recovery stage, electric signal reached similar values to those found in the basal period.

All raw data obtained from the acid-based metabolites and readings of the electrochemical sensors are detailed in Supplementary Tables S1–S5.

### 3.3. Fetal Hemodynamics

Table 1 depicts Doppler results of the different phases of the study available for six animals. In the 50% occlusion stage, only UV diameter presented a significant decrease in comparison with basal stage. The most relevant changes were observed in the 100% occlusion stage, where UV blood flow velocity and UV diameter presented a significant decrease.

### 3.4. Correlation Results

Correlation results are shown in Table 2. A high correlation was observed between the oxygen metabolite with the current signal detected by the oxygen sensor, although without reaching statistical significance. The correlation between pH and the electrochemical signal was weaker. On the contrary, a significant correlation between sensor signaling and Doppler evaluation was observed in the pH sensing. The correlation was significant especially with the UAPI and the UV blood flow.

### 3.5. Histology

**Figure 4:**
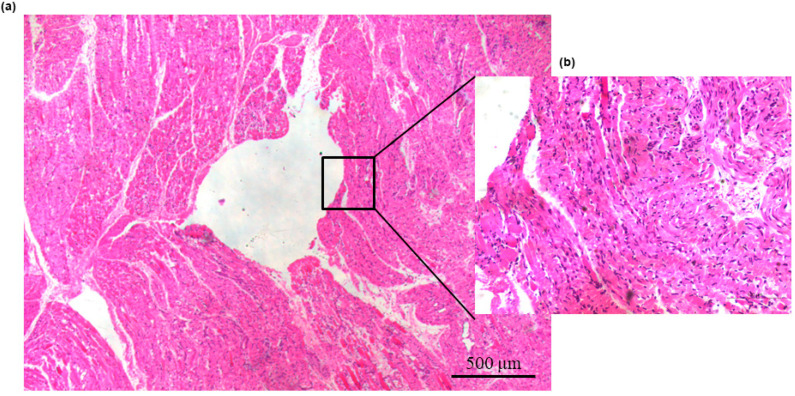
Images of (**a**) hematoxylin/eosin and (**b**) Masson trichrome staining of sites of implantation. (**a**) Normal muscular parenchyma delimitating the area of implantation without any inflammatory reaction at 5×; (**b**) Normal muscular parenchyma delimitating the area of implantation without any deposit of collagen fibers at 20×.

Muscular tissue surrounding the implantable sensor insertion area showed no signs of alteration around the site of implantation. Normal muscular parenchyma, with neither inflammatory reaction nor deposition of collagen fibers, was observed (Figure 4).

## 4. Discussion

In this study, we report for the first time a capability to monitor prenatal asphyxia continuously with specific miniaturized multiparametric electrochemical sensors for pH and pO_2_ in fetal sheep.

### 4.1. Acid-Based Metabolites

Our results of partial occlusion (50%) are in accordance with other models with partial UCO in fetal sheep at 130 days of gestation [16], with a decrease of 4 units in the arterial pO_2_ and 0.04 in pH. These modest falls in the acid-based metabolites contrasted with the marked alterations obtained in the 100% occlusion stage. In comparison with the basal level, significant decreases of arterial pO_2_ (22.64 mmHg to 11.73 mmHg) and fetal pH (7.24 to 6.86) were obtained. These pronounced alterations are comparable with other severe UCO models in fetal sheep between 124 and 131 days of gestation [17,18,19]. 

When comparing the pattern of changes for each metabolite, the most pronounced and fastest change was the decrease in the pO_2_, whereas pH alterations were less pronounced and more gradual. During the recovery, basal values were only reached in pO_2_ whereas changes in the remaining parameters did not fully revert to the basal values. This can be explained by the fact that changes in pH, lactate, HCO_3_^−^, and K+ are produced by the cellular metabolism in response to the decrease in oxygen levels in the blood. This response includes several changes at the cellular level making it a slower and more gradual response in comparison with the oxygen levels [20]. In fact, in some of the cases, the pH persisted below fetal viability (ranging around 6.69 and 6.77) leading to fetal death. These differences in the pattern of changes for each parameter have already been documented in previous studies with similar methodology [17,18,19].

### 4.2. Electrochemical Sensors 

Overall, the oxygen sensor detected pO_2_ changes induced by the occlusion protocol in the umbilical cord. The signal of the oxygen sensor increased in inverse proportion to the decrease in pO_2_, suggesting a good performance of the sensor in detecting pronounced changes in pO_2_. Additionally, in the recovery stage, the electrochemical signal returned to basal levels following the changes obtained in the blood test analyses. Previous studies with electrochemical sensors were designed similar to those used in this study demonstrated different sensibilities. In an adult rabbit model with a ventilatory hypoxia induction, the pO_2_ electrochemical sensor inserted in the skeletal muscle presented lower sensibility in comparison with the sensibility described here [12]. These differences may be explained with different reasons. Firstly, the animal model used may have an influence. In the previous study, acute ventilatory hypoxia was induced in an adult rabbit, in contrast with the UCO model induced in fetal sheep presented here. Secondly, basal oxygen levels differ between adults and fetuses since the amount of oxygen that reaches the fetal circulation is reduced, meaning that even small changes of oxygen level can cause a change from normoxia to hypoxia. Thirdly, differences in oxygen-hemoglobin and oxygen-myoglobin dissociation curves between fetuses and adults can explain the difference in function of the sensors. Fetal hemoglobin and myoglobin from skeletal muscle tissue have a higher affinity for oxygen than adult hemoglobin and myoglobin, becoming more saturated at the same oxygen levels [21,22]. Finally, differences in the design of the sensors with longer wires may also partially explain these results.

Regarding pH sensor, the pH sensor detected the pattern of pH blood changes induced by the umbilical occlusion protocol, although its sensibility was weaker in comparison with the oxygen sensor. During the occlusion period, an increase in the signal from the pH electrochemical sensor was obtained following the pattern of pH reduction in the blood test analysis, suggesting a good functionality of the sensor. Regarding the lower sensibility in comparison with the oxygen sensor, this could be due to the lower percentage of pH change during the UCO in comparison with the pO_2_ values. In addition, the decrease in pH due to the lack of oxygen requires the activation of the cellular anaerobic metabolism with the subsequent lactic acid formation. In this sense, tissue acidosis is more delayed and slower than the marked changes in the oxygen levels that only depend on the blockage of the oxygenated blood flowing through the umbilical cord. In the recovery stage, although the pH began to progressively increase, the signal measured with the electrochemical sensor implanted in the muscular tissue dropped more steeply during the first 10 min, reaching similar values to the basal period. On the other hand, standard pH equipment used to analyze blood did not reach baseline values during the study. This could be because acidosis produced in the tissue by cells affected by hypoxia takes some time to reach the blood flow, producing a delay in the response in blood-based measurement. Finally, as observed with the oxygen sensor, the sensibilities observed for the pH sensing differ from a previous study [12]. Again, these differences may be explained by the same three reasons as discussed above on the differences observed with the oxygen sensor.

Finally, the most relevant correlations were detected between Doppler parameters and electrochemical sensing, especially between UAPI and UV blood flow with the pH electrochemical sensor signal. On the contrary, a weaker correlation was detected between Doppler parameters and pO_2_ levels. Along this line, previous papers detected a significant association between lower levels of pH and Doppler changes [23]. Both pH and Doppler changes reflect a more stationary stage in the hypoxia-acidosis process, whereas pO_2_ changes are more dynamic and reversible [24].

### 4.3. Strengths and Limitations

Two of the main strengths of this study are the size of the electrochemical sensors and the added value of their multiparametric design. Their minimally invasive size means they can potentially be inserted by a needle that can be later withdrawn, leaving the sensor in the fetal skeletal muscle. Additionally, the multiparametric electrochemical sensors offer dynamical information regarding the different stages of tissue ischemia. Thanks to the oxygen sensor the device could detect an initial lack of oxygen, whereas the pH sensor may detect the tissue’s response to anaerobic conditions resulting in tissue acidosis. This information would be of value in clinical practice since it would give reliable information regarding the metabolic status of the fetus in real-time. Finally, the transferability of this research to clinics is high since the animal model used (pregnant sheep model) has been extensively used due to its similar fetal size and suitability to reproduce well the fetal hypoxic conditions found in human pregnancy [25].

This study has also some limitations. First, the electrochemical sensors studied here were able to detect changes at the 100% occlusion stage, while the acid-base changes were more pronounced. The detection of acid-based metabolites in fetal tissue is a challenge itself as the amount of oxygen that reaches the fetal circulation is reduced in comparison with adults, meaning that the difference in oxygen concentration between basal and hypoxia is very small [26]. In this regard, future directions of this work would be to improve the sensibility of these miniaturized electrochemical sensors in order to be able to detect more subtle hypoxia-acidosis changes such as those that occur in the 50% occlusion stage, more comparable to mild and chronic hypoxia-ischemia episodes. Finally, we did not assess long-term functionality testing; further studies including follow-up evaluation would be required. Finally, future sensor design focused on the telemetric system would give more functions to the whole system allowing real-time monitoring.

## 5. Conclusions

This study provides the first evidence showing the application of miniaturized multiparametric electrochemical sensors, capable of detecting changes in oxygen and pH in skeletal muscular tissue in fetal sheep. The use of such devices would open new applications in the field of fetal monitoring of pregnancies at high risk of fetal hypoxia-acidosis. Further research is required to advance in the adaptation and refinement of these electrochemical sensors to render them suitable for clinical testing. 

## Figures and Tables

**Table 1:** Evolution of Doppler hemodynamics during the UCO protocol.

Stage	Basal	50% Occlusion	100% Occlusion	Recovery
FHR (bpm)	135.8 (5.50)	129.3 (13.68)	116.8 (21.79)	115.7 (12.83)
UAPI	1.35 (0.23)	1.48 (0.23)	3.09 (1.24)	0.99 (0.06)
DVPI	0.52 (0.09)	0.46 (0.06)	1.79 (0.47)	2.92 (2.37)
UV flow velocity (cm/s)	16.38 (3.61)	18.09 (6.79)	−17.70 (10.96) *	19.78 (7.96)
UV diameter (mm)	5.77 (0.41)	3.79 (0.22) **	2.95 (0.51) ***	5.77 (0.31)

Statistical significance was declared when * *p* < 0.05, ** *p* < 0.01 and *** *p* <0.001 between basal and each stage. Values are mean and standard error of mean. FHR—fetal heart rate; UAPI—pulsatility index of the umbilical artery; DVPI—pulsatility index of the ductus venosus; UV—umbilical vein.

**Table 2:** Correlation results.

(**a**) Correlation between electrochemical sensing results and pO_2_ and pH results				
**Electrochemical Signal** **(nA or mV)**	**pO_2_**		**pH**	
nA	0.82		—	
mV	—		0.37	
(**b**) Correlation between electrochemical sensing results and Doppler results				
**Electrochemical Signal** **(nA or mV)**	**UAPI**	**DVPI**	**UV** **Diameter**	**UV Blood Flow Velocity**
nA	0.77	0.13	0.71	0.79
mV	0.99 *	0.01	0.68	0.95 *

Data are expressed as r squared. Statistical significance was declared when * *p* < 0.05. “—” no correlation possible; UAPI—pulsatility index of the umbilical artery; DVPI—pulsatility index of the ductus venosus; UV—umbilical vein.

## Data Availability

All data generated or analyzed during this study are included in this published article [and its Supplementary Materials].

## Supplementary Materials

The following are available online at https://www.mdpi.com/article/10.3390/biomedicines9101344/s1, Figure S1: Scheme of miniaturized electrochemical sensors: pH and pO_2_; Table S1: Acid-based metabolite results during the umbilical cord occlusion protocol; Table S2: Electric current measured by oxygen electrochemical sensors during the umbilical cord occlusion protocol; Table S3: Oxygen and pH results during the umbilical cord occlusion protocol from the animals used for oxygen electrochemical sensor evaluation; Table S4: Electric potential measured by pH electrochemical sensors during the umbilical cord occlusion protocol; Table S5: Oxygen and pH results during the umbilical cord occlusion protocol from the animals used for pH electrochemical sensor evaluation.
