# Supplementary material for: Miniaturized Electrochemical Sensors to Monitor Fetal Hypoxia and Acidosis in a Pregnant Sheep Model
Source: Biomedicines. 2021 Sep 28;9(10):1344. doi: 10.3390/biomedicines9101344 (PMC8533400; doi:10.3390/biomedicines9101344)
Supplement: Supplementary file 1 [file biomedicines-09-01344-s001.zip › biomedicines-1386471-supplementary.pdf]

biomedicines

MDPI

Supplementary Materials

Miniaturized Electrochemical Sensors to Monitor Fetal Hypoxia and Acidosis in a Pregnant Sheep Model

Míriam Illa [1,2,*,†], Laura Pla [1,†], Sergio Berdún [1], Mònica Mir [3,4,5], Lourdes Rivas [4], Samuel Dulay [4], Nicole Picard-Hagen [6], Josep Samitier [3,4,5], Eduard Gratacós [1,2,7,8], Elisenda Eixarch [1,7,8]

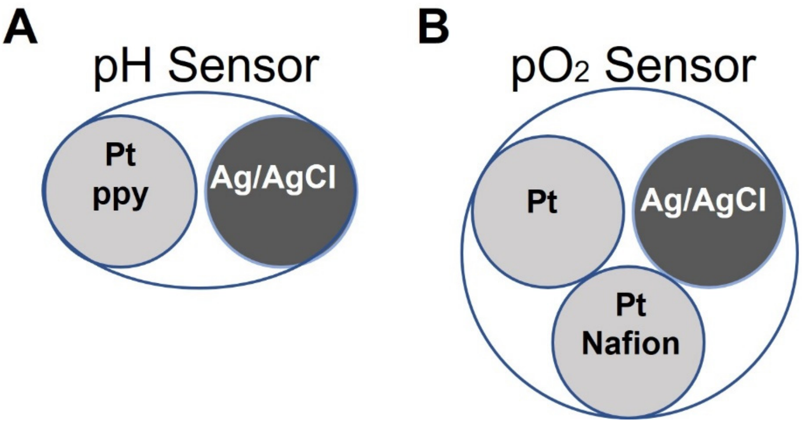

Figure S1. Scheme of miniaturized electrochemical sensors: pH and pO2. (**A**) Scheme of the pH sensor set up containing a platinum working electrode modified with a polypyrrole membrane specific to protons and a silver/silver chloride reference electrode. (**B**) Scheme of the oxygen sensor set up containing a platinum counter electrode, a silver/silver chloride reference electrode and a Nafion modified platinum working electrode.

Table S1. Acid-based metabolites results during the umbilical cord occlusion protocol.

Time		0	10	20	30	35	40	50	60
		BASAL		50% OCCLUSION		100% OCCLUSION		RECOVERY	
pO_2 (mmHg)	nº values	**3**	**7**	7	6	5	7	4	4
	Mean	22.70	22.64	17.57	20.95	9.62	11.73	24.70	23.38
	SD	3.58	3.14	5.67	2.57	4.39	4.81	5.51	2.45
	SEM	2.07	1.19	2.14	1.05	1.97	1.82	2.75	1.22
pH	nº values	3	7	7	6	5	7	4	4
	Mean	7.27	7.24	7.13	7.17	6.94	6.86	6.98	7.06
	SD	0.03	0.04	0.08	0.07	0.09	0.1	0.07	0.08
	SEM	0.01	0.01	0.03	0.03	0.04	0.04	0.03	0.04
Lactate (mmol/L)	nº values	4	7	7	6	5	7	4	4
	Mean	5.20	5.86	8.35	6.84	7.84	9.12	11.96	11.22
	SD	2.16	1.78	3.28	2.52	5.58	4.34	3.11	2.76
	SEM	1.08	0.67	1.24	1.03	2.49	1.64	1.55	1.38
HCO_3^- (mmol/L)	nº values	4	7	7	5	5	7	4	4
	Mean	26.25	24.93	24.50	24.96	21.54	19.76	19.18	18.50
	SD	3.35	3.03	3.69	2.77	5.96	3.40	3.63	1.74
	SEM	1.68	1.14	1.39	1.24	2.67	1.28	1.82	0.87
K^+ (mmol/L)	nº values	4	7	7	6	5	7	4	4
	Mean	3.98	3.80	4.20	4.42	4.56	4.53	4.13	3.90
	SD	0.39	0.42	0.40	0.63	1.35	1.11	0.46	0.26
	SEM	0.19	0.16	0.15	0.26	0.60	0.42	0.23	0.13

Table S2. Electric current measured by oxygen electrochemical sensors during the umbilical cord occlusion protocol.

Time		0	10	20	30	35	40	50	60
		BASAL		50% OCCLUSION		100% OCCLUSION		RECOVERY	
Electric current (nA)	nº values	2	5	7	6	7	3	5	3
	Mean	-3.68	-4.01	-3.63	-3.10	-2.31	-2.75	-4.31	-2.50
	SD	0.35	0.85	0.66	0.69	1.12	1.54	2.36	0.54
	SEM	0.25	0.38	0.25	0.28	0.42	0.89	1.06	0.31

Table S3. Oxygen and pH results during the umbilical cord occlusion protocol from the animals used for oxygen electrochemical sensor evaluation.

Time		0	10	20	30	35	40	50	60
		BASAL		50% OCCLUSION		100% OCCLUSION		RECOVERY	
pO_2 (mmHg)	nº values	3	6	6	5	5	6	4	4
	Mean	22.83	22.15	17.67	20.12	9.620	10.45	24.70	23.38
	SD	3.75	3.13	6.21	1.75	4.39	3.75	5.51	2.45
	SEM	2.16	1.28	2.53	0.78	1.97	1.53	2.75	1.22
pH	nº values	3	6	6	5	5	6	4	4
	Mean	7.24	7.23	7.13	7.17	6.94	6.84	6.98	7.06
	SD	0.03	0.02	0.09	0.07	0.09	0.09	0.07	0.08
	SEM	0.02	0.01	0.04	0.03	0.04	0.04	0.03	0.04

Table S4. Electric potential measured by pH electrochemical sensors during the umbilical cord occlusion protocol.

Time		0	10	20	30	35	40	50	60
		BASAL		50% OCCLUSION		100% OCCLUSION		RECOVERY	
Electric potential (mV)	nº values	5	9	10	7	10	3	9	6
	Mean	237.91	231.63	225.0	246.30	273.41	298.04	214.35	220.42
	SD	78.65	65.10	27.15	41.12	55.43	56.30	21.85	31.54
	SEM	35.17	21.70	8.585	15.54	17.53	32.51	7.284	12.88

Table S5. Oxygen and pH results during the umbilical cord occlusion protocol from the animals used for pH electrochemical sensor evaluation.

Time		0	10	20	30	35	40	50	60
		BASAL		50% OCCLUSION		100% OCCLUSION		RECOVERY	
pO_2 (mmHg)	nº values	3	6	6	5	4	6	6	5
	Mean	23.70	23.30	19.10	21.34	9.73	11.95	17.83	17.28
	SD	3.08	2.86	4.36	2.66	5.07	5.23	6.41	7.53
	SEM	1.78	1.17	1.78	1.19	2.53	2.14	2.62	3.37
pH	nº values	3	6	6	5	4	6	6	5
	Mean	7.25	7.23	7.15	7.19	6.97	6.87	6.88	6.92
	SD	0.04	0.04	0.06	0.05	0.06	0.11	0.12	0.19
	SEM	0.03	0.02	0.02	0.02	0.03	0.04	0.05	0.09
